# Detecting eyes with high risk of angle closure among apparently normal eyes by anterior segment OCT: a health examination center-based model

**DOI:** 10.1186/s12886-022-02739-7

**Published:** 2022-12-28

**Authors:** Sigeng Lin, Ying Hu, Cong Ye, Nathan Congdon, Ruirong You, Shanshan Liu, Chi Liu, Fan Lv, Shaodan Zhang

**Affiliations:** 1 The Eye Hospital, School of Ophthalmology and Optometry, Wenzhou Medical University, No.270 Xueyuanxi Street, Lucheng District, Wenzhou, 325027 Zhejiang China; 2 Glaucoma Research Institute, Wenzhou Medical University, Wenzhou, China; 3 National Clinical Research Center for Ocular Diseases, Wenzhou, China; 4 Department of Ophthalmology, The Forth People’s Hospital of Shenyang, Huanggu District, NO. 20 Huanghenan Street, Shenyang, 110031 Liaoning China; 5 Centre for Public Health, Queen’s University Belfast, Belfast, UK; 6 Zhongshan Ophthalmic Center, Sun Yat-Sen University, Guangzhou, China; 7 Orbis International, New York, NY USA

**Keywords:** Narrow angle, Screening, Anterior segment OCT, Health examination center

## Abstract

**Background:**

The main barriers keeping individuals with high-risk of angle closure from seeking eye-care service are the absence of both disease awareness and convenient and low-cost access to the ocular health care system. Present study described the efficacy of a health examination center-based screening model designed to detect eyes with high risk of angle closure (HRAC) among healthy individuals using anterior segment optical coherence tomography (AS-OCT).

**Methods:**

From March 1 to April 30, 2017, consecutive individuals aged ≥ 40 years undergoing routine physical examinations at a health examination center were invited to enroll. Presenting visual acuity (PVA), intraocular pressure (IOP) measurement, non-mydriatic fundus photography and AS-OCT were performed by three trained nurses. Participants with PVA < 6/12 in the better-seeing eye, IOP ≥ 24 mmHg, or abnormal fundus photography in either eye were referred to the outpatient clinic, but not included in the analysis. Eyes with HRAC were defined as having trabecular-iris angle < 12 degrees in ≥ 3 quadrants. Configuration of the iris was classified into flat, bowing, bombe, thick peripheral iris and mixed mechanism.

**Results:**

Altogether, 991 participants (77.3%) with readable OCT images (mean age 55.5 ± 9.0 years; 58.4% men) were included. HRAC was diagnosed in 78 eyes (7.9%, 61.3 ± 8.2 years, 41.0% men). The prevalence of HRAC increased with age (*p* < 0.001) and was much higher among women (11.2%) than men (5.5%) (*p* = 0.001). The mixed mechanism iris configuration was most common among eyes with HRAC (37/78, 47.4%).

**Conclusion:**

HRAC is prevalent among asymptomatic Chinese adults undergoing routine health screening. Health examination center-based eye screening with AS-OCT administered by non-specialists may be a good model to screen narrow angles in the population at large.

## Background

Glaucoma is the leading cause of irreversible blindness, affecting around 80 million people worldwide [1]. More than half of people with primary angle closure glaucoma (PACG) live in Asia. A high rate of blindness from PACG has been reported in China [2–5], resulting in a heavy burden both on affected families and society at large [6]. Early detection and appropriate prophylactic interventions can prevent eyes with angle closure from progressing to PACG [7]. Previously, we reported that combined population screening for primary open angle glaucoma (POAG) and PACG among Chinese adults is likely to be cost-effective due to the low labor costs, high prevalence and blindness rate of PACG, and low opportunistic detection rates [8]. However, the main barriers keeping individuals with high-risk of angle closure from seeking eyecare service are the absence of both disease awareness and convenient and low-cost access to the ocular health care system.

Health examination centers are well-established public health delivery system in China, with nearly 10,000 centers across China covering a population of 700 million at present [9]. People come to these centers for a general evaluation of their health status, either individually or as organized by their employers. We previously reported a model of integrating glaucoma screening into general health examinations [10]. This model provides opportunities for screening of vision-threatening eye diseases among the population at large. Here, we report the efficacy of health examination center-based screening for eyes with high risk of angle closure among healthy, asymptomatic persons, by using anterior segment optical coherence tomography (AS-OCT).

## Methods

This study was conducted in accordance with the tenets of the Declaration of Helsinki. The Ethics Committee of the Fourth People's Hospital of Shenyang approved this cross-sectional study (SYSY_2017006) and determined that informed consent was not required, as all data were collected and analyzed in de-identified fashion.

## Eye disease screening

All participant (age ≥ 40) presenting to the Health Examination Center of the Fourth People's Hospital of Shenyang from March 1 to April 30, 2017 were invited to participate. Ocular examinations, including presenting visual acuity (PVA), non-contact tonometry (CT-1P Computerized Tonometer, Topcon Ltd, Tokyo, Japan), fundus photography (Canon CX-1, Tokyo, Japan) and AS-OCT (TOMEY Corporation Japan, Nagoya, Japan) were performed by three trained nurses. Glaucoma was defined according to the classification system described by the International Society for Geographical and Epidemiological Ophthalmology (ISGEO) [11]. Participants with PVA < 6/12 in the better-seeing eye, IOP ≥ 24 mmHg, or abnormalities on fundus photography in either eye were referred to the ophthalmology outpatient clinics, where their angle status will be further assessed by the confirmative examinations. Therefore, these individuals were not included in present study, in which we focus exclusively on asymptomatic persons.

## AS-OCT imaging and measurements

First, to set a criteria of high risk of angle closure (HRAC) on AS-OCT images, we compared the AS-OCT images with the gonioscopy examination in the same eye in patients with clinically diagnosed primary angle closure suspects (PACS). PACS were defined as not having visible posterior trabecular meshwork in two or more quadrants examined by noncompression gonioscopy, IOP ≤ 21 mmHg, absence of peripheral anterior synechiae or glaucomatous optic neuropathy. Only images in the quadrants classified as Scheie grading N3 by gonioscopy were analyzed. Scheie grading N3 was defined as only anterior 1/3 trabecular meshwork visible. Finally, 97 AS-OCT images (TOMEY Corporation Japan, Nagoya, Japan) from 49 eyes of 32 PACS patients (18 females and 14 males, with a mean age of 61.7 ± 8.9 years) were included. A mean trabecular-iris angle (TIA) of 12.1 ± 5.8 degree was observed in these AS-OCT images. So TIA of 12 degree was used for the diagnosis of HRAC in the following screening scheme.

During screening, AS-OCT imaging was performed in a seated position under standardized dark conditions with ambient illumination below 5 lx. During imaging, the eyelids were gently retracted, taking care to avoid inadvertent pressure on the globe. Two images were exported in Joint Photographic Experts Group (JPEG) format per eye: one oriented along the horizontal (temporal-nasal) meridian and the other along the vertical (superior-inferior) meridian. Images with poor quality due to eye movement or lid overlapping were excluded. AS-OCT images of the right eye were analyzed, unless images of at least two quadrants were un-identifiable, in which case the images of the left eye was used.

**Fig. 1:**
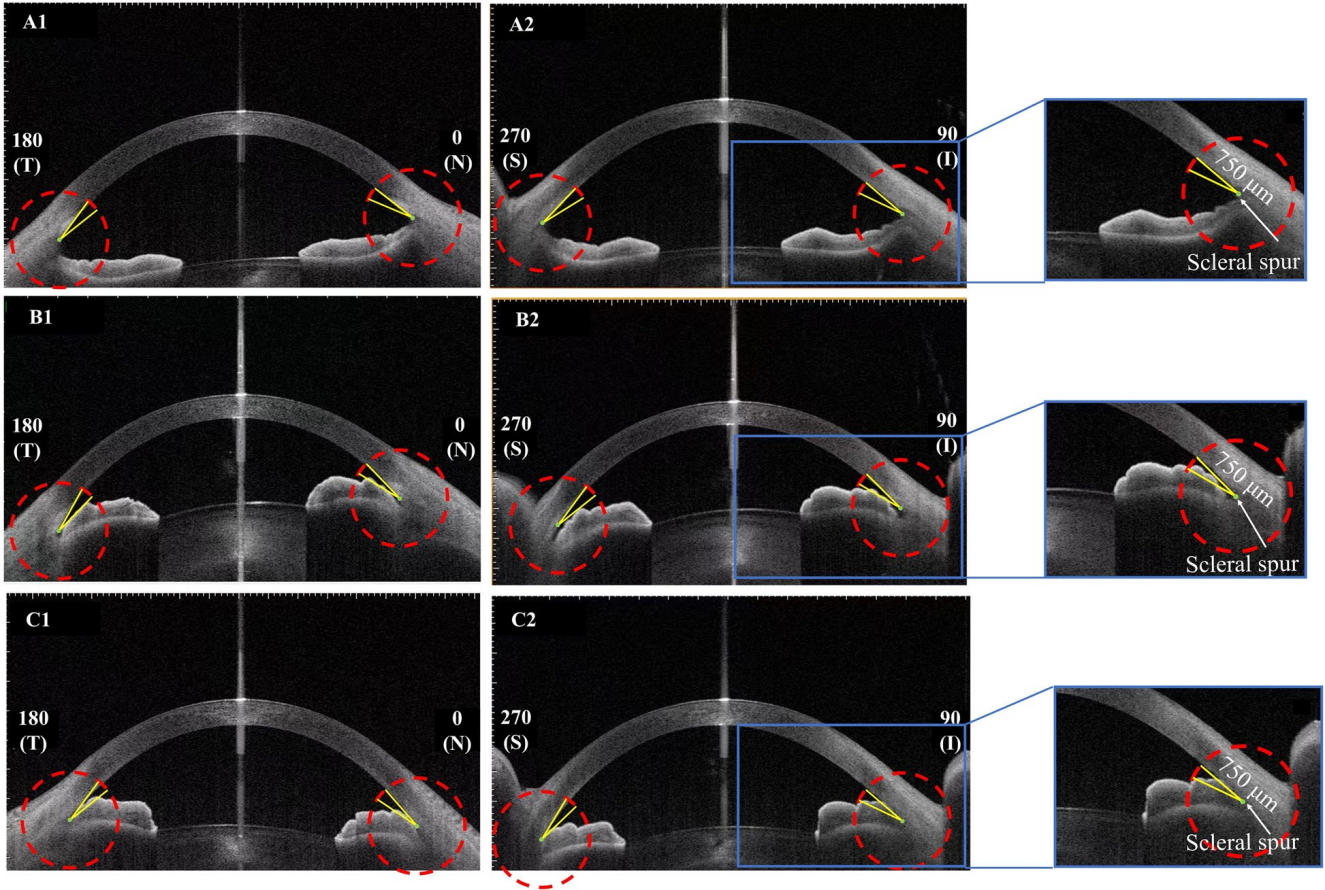
Classification of the anterior chamber angles with a preset 12-degree angle. With the apex being located in the iris recess, one arm of the angle was adjusted to pass through a point on the trabecular meshwork 750 μm from the scleral spur. The angle was classified into: open (**A**), narrow (**B**) and closed (**C**) according to the location of the other arm of the preset angle. Four quadrants in the same eye were assessed. Eyes with narrow and/or closed angle in three quadrants and above were defined as at high-risk of angle closure (HRAC)

**Fig. 2:**
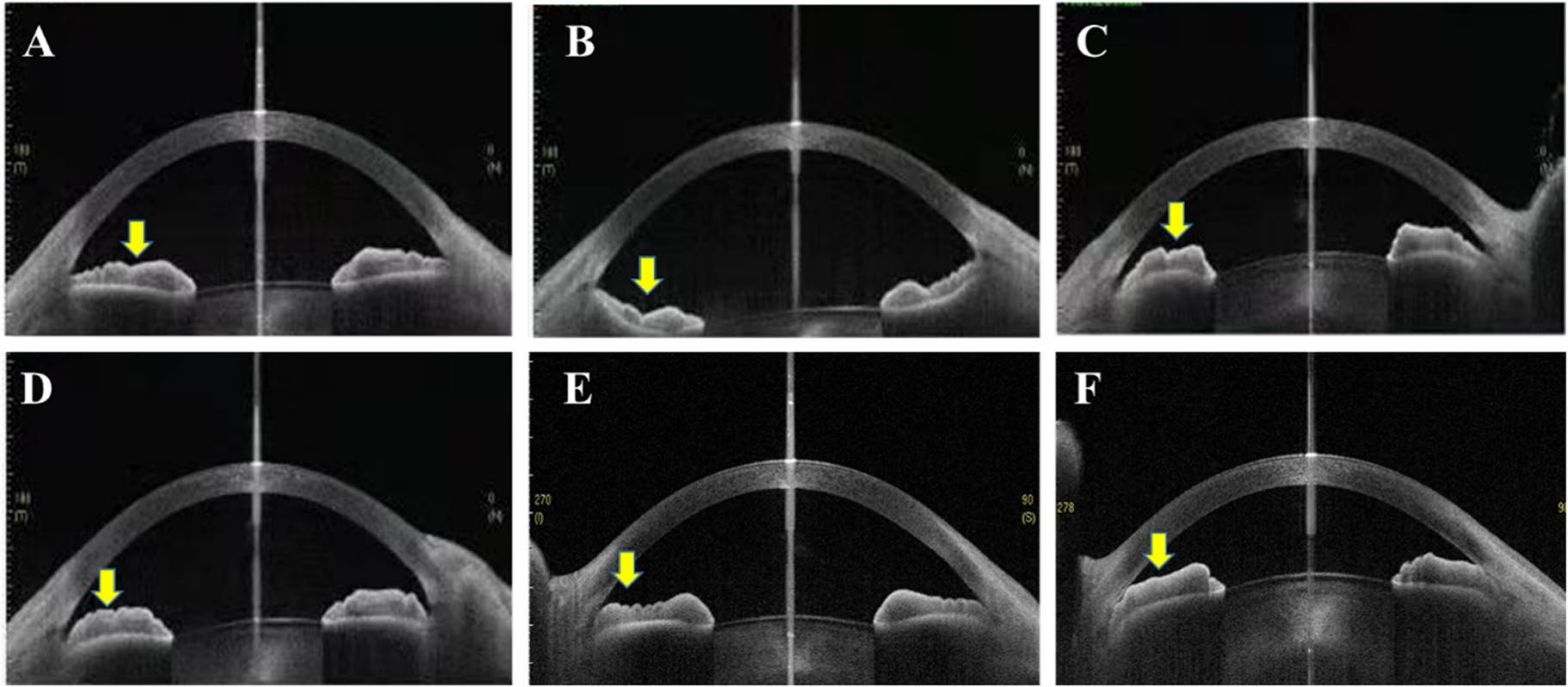
Iris configuration was classified into flat (**A**), bowing (**B**), bombe (**C**), thick peripheral iris (**D** and **E**) and mixed (**F**, iris bombe and thick peripheral iris)

The AS-OCT images in four quadrants were assessed at a preset of 12 degrees: with the apex being located in the iris recess, one arm of the angle was adjusted to pass through a point on the trabecular meshwork 750 μm from the scleral spur. The angle was classified into open, narrow and closed according to the location of the other arm of the preset angle (open: anterior surface of peripheral iris fallen out of the other arm of the preset angle; narrow: anterior surface of peripheral iris fallen into the other arm of the preset angle; and closed: trabecular meshwork and peripheral iris were located appositionally) (Fig. 1 A-C). Four quadrants in the same eye were assessed. Eyes with narrow and/or closed angles in ≥ 3 quadrants were considered as being at HRAC. The iris configuration was classified into flat, bowing, bombe, thick peripheral iris, and mixed. If two or more configurations were observed in the same quadrant or in different quadrants of the same eye, a mixed configuration was defined. (Fig. 2, A-E). Images were evaluated independently by two glaucoma specialists (SDZ and CY). When there was disagreement, a third analyst was consulted (CL). Individuals diagnosed as HRAC were suggested to receive definitive examinations at the ophthalmology outpatient clinics. Telephone interviews were also conducted to give a brief introduction of their condition and basic knowledge about primary angle-closure diseases (PACD).

## Statistical analysis

Statistical analysis was performed using SPSS (version 18.0; SPSS, Inc., Chicago, IL, USA). Continuous and categorical data were presented as mean ± standard deviation and using counts respectively. Continuous data were compared using the Student’s t-test or Mann–Whitney U test, and categorical data was compared using the Chi-square test. The significance level was set at 0.05.

## Results

**Fig. 3:**
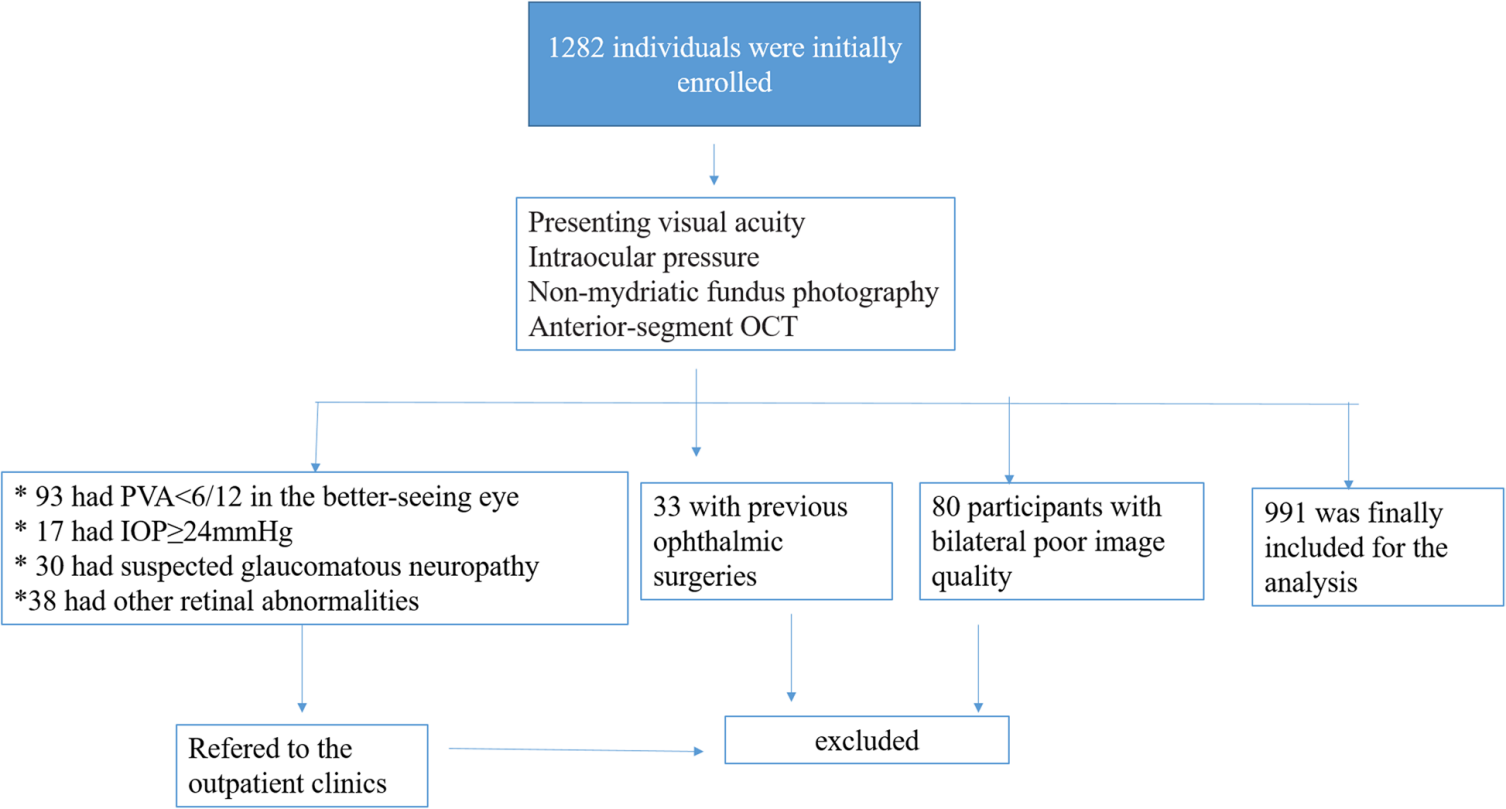
Study flow chart

A total of 1282 participants were initially enrolled. Eighty participants (6.2%) were excluded from the analysis due to bilateral poor image quality. In addition, 93 individuals (7.3%) had PVA < 6/12 in the better-seeing eye, 17 (1.3%) had IOP ≥ 24 mmHg, 30 (2.3%) had suspected glaucomatous neuropathy, 38 (3.0%) had other retinal abnormalities in the fundus photograph, and 33 (2.6%) with previous ophthalmic surgeries were also excluded. Ultimately, 991 images from 991 participants (58.4% men, mean age 55.5 ± 9.0 years, range, 40–84 years) were included in the analysis (Fig. 3). Among these participants, 78 (7.9%, 32 men, 46 women) were defined as HRAC, including 2 cases of appositional angle closure (Table 1). The age- and gender-specific prevalence rates are shown in Table 2. The prevalence of HRAC increased with age (*p* < 0.001). The mean age of persons with HRAC (61.3 ± 8.2 years) was significantly older than for those without (55.0 ± 8.9 years) (*p* < 0.001). The prevalence of HRAC among women (11.2%) was significantly higher than that among men (5.5%, *p* = 0.001, Table 2). Eyes with HRAC predominantly had a mixed iris configuration (37/78, 47.4%), followed by thick peripheral iris (22/78, 28.2%) and iris bombe (19/78, 24.4%) (Table 2).

**Table 1:** Characteristic and iris configuration of participants with and without high-risk of angle closure (HRAC)

Characteristics	With High Risk of Angle-Closure (*N* = 78)	Without High-risk of Angle-Closure (*N* = 913)	*P* value
Mean age, years (± standard deviation)	61.3 ± 8.2	55.1 ± 8.9	** < 0.001**
Male gender (%)	32 (41.0)	547 (59.9)	**0.001**
Mean presenting visual acuity (logMAR)	0.15 ± 0.16	0.16 ± 0.24	0.161
Mean IOP (mmHg)	16.1 ± 2.4	16.7 ± 2.8、	0.082
**Iris configuration (n, %)**			
Flat	0(0)	666 (72.9)	** < 0.001**
Bowing	0(0)	51 (5.6)	
Iris bombe	19 (24.4)	15 (1.6)	
Thick peripheral iris	22 (28.2)	118 (12.9)	
Mixed	37(47.4)	63 (6.9)

**Table 2:** Distribution of eyes with high risk of angle closure by age and gender

	Men	Women	Total	*P* value
Age, years				
40–49	1 /165(0.6%)	8/148 (5.4%)	9/313 (2.9%)	**0.011**
50–59	6/190 (3.2%)	16/150 (10.7%)	22/340 (6.5%)	**0.005**
60–69	18/186 (9.7%)	18/98(18.4%)	36/284 (12.7%)	**0.036**
≥ 70	7/20 (35.0%)	4/16 (25.0%)	11/36 (30.1%)	0.584
Total	32/579 (5.5%)	46/412(11.2%)	78/991 (7.9%)	**0.001**
*P* value	** < 0.001**	**0.004**	** < 0.001**	

## Discussion

Easy and convenient access, fast and non-invasive examinations not requiring specialist care, accurate diagnosis, and cost-effectiveness of the screening are key factors for a scalable model of glaucoma screening and referral suitable for low-resource settings. In the current study, we described the efficacy of a semi-quantitative assessment of the anterior chamber angle by AS-OCT for the detection of eyes with high-risk of angle closure among asymptomatic persons presenting for general screening at a health examination center. Compared to persons with high IOP and poor visual acuity, that may lead to obvious symptoms prompting them to seek medical care, those with high risk of narrow angles, good visual acuity and normal IOP will likely lack any symptoms until and unless they develop acute or chronic angle closure. The health examination center screening model provides an opportunity to detect these asymptomatic individuals and prevent severe visual impairment by intense follow-up and prophylactic management when necessary. AS-OCT is quick and non-invasive, and combined with a semi-quantitative assessment as described here, can be readily performed by non-specialists after modest training, or by artificial intelligence software in the near future [12, 13].

Several methods have been described for population-based screening of primary angle closure disease (PACD), including Van Herick assessment, gonioscopy, ultrasound biomicroscopy (UBM) and AS-OCT [5, 14, 15]. The Van Herick test is convenient and quick, but the low specificity and positive predict value (PPV) are likely to limit its utility as a population-based screening tool, even in populations with high prevalence of PACD [15]. Currently, gonioscopy is still the gold standard for diagnostic angle assessment. However, this test is subjective, semi-invasive. It requires specialized training and relies on good patient cooperation, which is not suitable for screening at large. UBM provides high-quality images for an objective evaluation of the anterior chamber angle, but does not overcome other disadvantages of gonioscopy as described above, which greatly limits its application in large populations as a routine examination. An ideal instrument for PACD screening should not require scarce trained ophthalmic resources, while being rapid, noninvasive, repeatable and easy to interpret [15]. AS-OCT imaging meets all these requirements, providing a fast, contactless and objective method for angle assessment. It is highly sensitive in detecting angle closure when compared with gonioscopy, and shows similar specificity and sensitivity to UBM in identifying narrow angles [16–20]. However, diagnostic accuracy and specificity of AS-OCT in defining narrow angle was only moderate compared with gonioscopy. What’s more, it still requires skilled personnel to interpret the results, which may limit its application by non-ophthalmologists among large populations, unless simple, objective, and reasonable diagnostic protocols are used.

In the present study, we defined HRAC as consisting of TIA < 12 degrees in at least three quadrants of the AS-OCT images. A preset angle of 12 degrees, with the apex being located in the iris recess and one arm passing through a point on the trabecular meshwork 750 μm from the scleral spur, was used for a quick semi-quantitative assessment of the angle in AS-OCT images. The reasons are as follows. First, in most studies, inability to visualize the posterior or pigmented trabecular meshwork in at least two or three quadrants has been used to define narrow angles, or PACS [21, 22]. To identify eyes at higher risk of angle closure, narrow angle in ≥ 3 quadrant was utilized as a criteria in present study. Second, we set the criteria of 12° for the diagnosis of HRCA through the comparison of AS-OCT with gonioscopy in clinically diagnosed PACS. Consistently, in Lan’s study, Shaffer grade 2 on gonioscopy, which is most commonly been used for defining a narrow angle [23], corresponded to a TIA of 11.67 ± 3.44° on AS-OCT images [24]. Third, Narayanaswamy et al. reported in a community-based study in Singapore that the angle opening distance (AOD) at 750 μm from the scleral spur (AOD750) was the most useful angle measurement for identifying individuals with gonioscopic narrow angles in gradable AS-OCT images [25]. However, no definite cut-off of AOD has been recommended for defining narrow angles at present. So, we used a distance of 750 μm from the sclera spur as a reference for the preset angle. With the scleral spur being recognized and labeled, the peripheral anterior chamber angle can be classified into HRAC vs non-HRAC by simply observing the location of the other arm of the preset angle relative to the anterior surface of the peripheral angle (as shown in Fig. 2). This simple-to-implement definition should greatly decrease the need for specialized medical training to interpret results, providing the basis for development of an artificial intelligence-aided diagnostic tool in the near future [13]. The sensitivity and specificity of this method still requires further investigation.

In present study, only four images from the superoinferior and temporonasal scans were utilized for the angle assessment. Nowadays, various commercial AS-OCT equipment provide 360-degree angle imaging. Sensitivity and specificity of a 3-dimensional deep-learning-based automated digital gonioscopy system for detecting narrow angle and peripheral anterior synechia was reported at 0.867 (0.838–0.895) and 0.878 (0.859–0.896), and 0.900 (0.714–1.000) and 0.890 (0.841–0.938), respectively [26]. Porporato et al. also reported that deep learning algorithm for 360-degree angle assessment of swept-source OCT exhibited good diagnostic performance for detection of gonioscopic angle closure [27]. However, results from the same research team also suggested that the single OCT scan at 80°-260°had the highest diagnostic performance and the 360° evaluation may not translate to better clinical utility for detection of gonioscopic angle closure [28]. Whatever, advancement of technology and artificial intelligence will definitely and greatly facilitate the screening at large in the near future.

In present study, we reported a prevalence of 7.9% and 10.2% for HRAC among unselected, asymptomatic individuals aged ≥ 40 years and ≥ 50 years, respectively (Table 2). These figures are lower than reported in the Handan Eye Study in north China for the prevalence of PACS (defined as posterior trabecular meshwork not visible for ≥ 180° on gonioscopy), 10.4% among those ≥ 40 years, and also for narrow angles (non-visible pigmented trabecular meshwork in three or more quadrants) in the Liwan eye study, 11.0% in a Chinese population aged 50 years and above [5, 29]. In present study, we mainly focus on improving the detection of HRAC and access to healthcare among asymptomatic persons. So, participants with PVA < 0.5 or abnormal fundus photography were excluded from the analysis in the present study. So, difference in the prevalence of narrow angle between studies may not only due to the different methods and definitions used, but may also be related to the persons enrolled. Nonetheless, the prevalence of HRAC is quite high in the asymptomatic eyes in Chinese adults, which should gain much attention from a public health’s respect.

It is important to consider the value of screening for narrow angles. In Chinese populations, the 5-year progression from primary angle closure suspect (PACS) to primary angle closure (PAC) or primary angle-closure glaucoma (PACG) is about 6.1% [30]. Using a screening cutoff of ≥ 6 clock hours of posterior trabecular meshwork not visible under non-indentation gonioscopy for the diagnosis of PACD, the Zhongshan Angle Closure Prevention (ZAP) Trial concluded that early detection and prophylactic intervention of for PACS may not have a large public health benefit, given the low observed 5-year prevalence of developing acute angle-closure or angle-closure glaucoma [31]. It is unclear that whether the results will be different if a criteria of ≥ 9 clock hours (270 degrees) is used. So, prophylactic iridotomy in high-risk eyes is still recommended currently to prevent affected individuals from experiencing acute angle closure attacks [32]. A strict, objective and reasonable criterion for defining high-risk angles is of special importance to avoid an undue burden on the healthcare system, particularly in countries like China with comparatively scarce resources. Noteworthy, in the Handan Eye study, 150 of 457 individuals with bilateral open angles at baseline (32.8%) developed PACS or PAC after 5 years [33]. This may be compared with the report of He and colleagues that approximately one in five people aged 50 years and older with open angles at baseline developed some form of angle closure over a 10-year period [34]. Since health examinations at most centers are repeated annually, our health examination center-based AS-OCT screening model provides ample opportunity for early detection, patient education and continuous monitoring of eyes with HRAC. It may ultimately improve the access to appropriate interventions among this population and help to reduce visual impairment associated with angle-closure glaucoma. Screening for open angle glaucoma, as described in our previous paper would proceed in parallel with this, and increase cost effectiveness of the detection process, as described in our prior models of combined screening [8, 10].

Limitations of the present study should be mentioned. First, our principal aim is to describe a new protocol for screening asymptomatic persons at high risk of narrow angles in a novel setting. The prevalence of HRAC in our study is not comparable with population-based studies and cannot be extrapolated to such settings. Secondly, 6.2% participants were excluded from the analysis due to bilateral poor image quality and did not receive other examinations for HRAC assessment in this screening scheme. Third, the high price of OCT equipment must be considered as a disadvantage of this method. In the present study, we did not perform a cost-effectiveness analysis of AS-OCT screening. Economy of scale by extending screening to large cohorts over time can presumably reduce the cost of screening. Fourth, outcomes to assess the impact on patient education, patient access and disease awareness should be further investigated. Fifth, high prevalence of PACDs, especially PAC and PACG, have been reported in patients with diabetes mellitus or/and systemic hypertension [35]. However, such diagnostic information, which could readily be obtained from the health examination records of screening, were not included in our current model.

## Conclusion

In conclusion, the prevalence of HRAC is high in asymptomatic, healthy Chinese adults. Health examination center-based screening facilitates greater contact with persons at high-risk of angle closure among the very large and growing cohort of individuals undergoing health examinations in China [10]. AS-OCT is a good option for the screening of PACDs in large populations. Developing semi-quantitative diagnostic tools with acceptable sensitivity and specificity, as well as artificial intelligence-assisted diagnostic systems in the near future, is a key task for implementing this screening model.

## Data Availability

All data supporting the findings are included in this article. The datasets during the study that are not presented in this article are available from the corresponding author on reasonable request.

## Abbreviations

AOD: Angle opening distance
AS-OCT: Anterior segment optical coherence tomography
HRAC: High risk of angle closure
ISGEO: International Society for Geographical and Epidemiological Ophthalmology
IOP: Intraocular pressure
JPEG: Joint Photographic Experts Group
PPV: Positive predict value
PVA: Presenting visual acuity
PAC: Primary angle closure
PACD: Primary angle closure disease
PACG: Primary angle closure glaucoma
PACS: Primary angle closure suspect
POAG: Primary open angle glaucoma
TIA: Trabecular-iris angle
UBM: Ultrasound biomicroscopy
