# Correction to: Detecting eyes with high risk of angle closure among apparently normal eyes by anterior segment OCT: a health examination center-based model

**DOI:** 10.1186/s12886-023-02849-w

**Published:** 2023-03-13

**Authors:** Sigeng Lin, Ying Hu, Cong Ye, Nathan Congdon, Ruirong You, Shanshan Liu, Chi Liu, Fan Lv, Shaodan Zhang

**Affiliations:** 1 The Eye Hospital, School of Ophthalmology and Optometry, Wenzhou Medical University, No.270 Xueyuanxi Street, Lucheng District, 325027 Wenzhou, Zhejiang China; 2 Glaucoma Research Institute, Wenzhou Medical University, Wenzhou, China; 3 National Clinical Research Center for Ocular Diseases, Wenzhou, China; 4 Department of Ophthalmology, The Forth People’s Hospital of Shenyang, Huanggu District, NO. 20 Huanghenan Street, 110031 Shenyang, Liaoning, China; 5 Centre for Public Health, Queen’s University Belfast, Belfast, UK; 6 Zhongshan Ophthalmic Center, Sun Yat-Sen University, Guangzhou, China; 7 Orbis International, New York, NY, USA

**Correction: BMC Ophthalmol 22, 513 (2022)**

10.1186/s12886-022-02739-7

Following publication of this article [1], it has been brought to our attention that Shaodan Zhang should have been denoted as corresponding author.

The author group has been updated above and the original article [1] has been corrected.
